# Digital video recording in trauma surgery using commercially available equipment

**DOI:** 10.1186/1757-7241-21-27

**Published:** 2013-04-11

**Authors:** Shokei Matsumoto, Kazuhiko Sekine, Motoyasu Yamazaki, Tomohiro Funabiki, Tomohiko Orita, Masayuki Shimizu, Mitsuhide Kitano

**Affiliations:** 1 Department of Trauma and Emergency Surgery, Saiseikai Yokohamashi Tobu Hospital, 3-6-1 Shimosueyoshi Tsurumi-ku, Yokohama-shi Kanagawa, 230-0012, Japan; 2 Department of Emergency Medicine, Saiseikai Chuo Hospital, 1-4-17 Mita Minato-ku, Tokyo, 108-0073, Japan

**Keywords:** Digital video recording, trauma surgery, education

## Abstract

**Introduction:**

Although videos of surgical procedures are useful as an educational tool, the recording of trauma surgeries in emergency situations is difficult. We describe an inexpensive and practical shooting method using a commercially available head-mounted video camera.

**Methods:**

We used a ContourHD 1080p Helmet Camera (Contour Inc., Seattle, Washington, USA.). This small, self-contained video camera and recording system was originally designed for easy videography of outdoor sports by participants.

**Results:**

We were able to easily make high-quality video recordings of our trauma surgeries, including an emergency room thoracotomy for chest stab wounds and a crush laparoptomy for a severe liver injury.

**Conclusion:**

There are currently many options for recording surgery in the field, but the recording device and system should be chosen according to the surgical situation. We consider the use of a helmet-mounted, self-contained high-definition video camera-recorder to be an inexpensive, quick, and easy method for recording trauma surgeries.

## Introduction

The number of trauma victims in developed countries has become much smaller during recent years. In addition, the success of non-operative management of many injuries has diminished the number of operations performed by trauma surgeons [1,2]. As a result, general surgeons in developed countries may lack experience in trauma surgeries. To address this problem, numerous training models have been developed that include the use of mannequins, live animals, and human cadavers [3]. However, there is no adequate substitute for the experience of real clinical cases, although the discussion of actual cases is an important aspect of surgical conferences. The cliché, “a picture is worth a thousand words,” is applicable here, especially with regard to the presentation of surgical procedures and the development of effective teaching aids. Therefore, the provision of high-quality surgical video recordings is desirable.

A variety of methods are currently available for capturing video of surgical procedures, such the operation of light mounted cameras, the use of head-mounted cameras (head-cams) designed for hospital environments, and laparoscopic cameras [4-7]. However, such medical video recording systems are typically very expensive and hence require considerable capital-investment. In addition, these systems tend not to be useful in trauma care scenarios because trauma personnel are too busy fulfilling the demands of their practice to pay attention to the recording of surgical video. Thus, video recordings of trauma surgeries such as an emergency thoracotomy in the emergency room and damage control surgery are rare. On the other hand, high-definition (HD) video-recording cameras that are light, self-contained, and inexpensive are now commercially available. Hours of HD video can thus be easily recorded, and just as easily played back and edited on a computer. Here, we describe an inexpensive and practical shooting method based on the use of a commercially available head-mounted video camera.

## Patients, materials, and methods

### Video acquisition and editing

**Figure 1:**
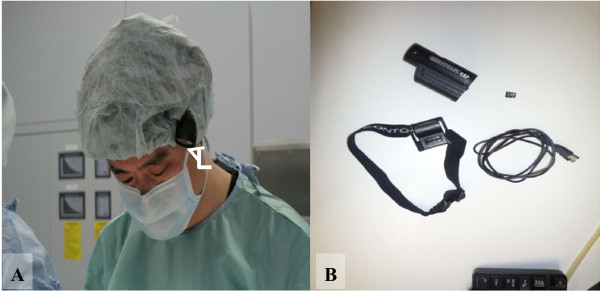
**Commercially available head-mounted camera (ContourHD, Contour Inc.,Seattle, Washington, USA.). A:** We wear the camera (white arrow) under a surgical cap. Because the device is cordless, video recording can be quickly and easily prepared. **B:** The equipment used in our study is shown. It comprises (clockwise): the head-mounted camera; 16GB microSD card; USB cable for transferring digital data to a computer; elastic headband camera mount.

Several commercially available head-cams currently can be purchased online for only a few hundred US dollars. We used the ContourHD 1080p Helmet Camera (Contour Inc., Seattle, Washington, USA. http://store.contour.com/), which can shoot crisp, smooth video at either 720p at 60 frames per second (fps) or 1080p at 30fps. This video camera was originally introduced for ease of use when recording outdoor sports. The supplied battery allows approximately 2 hours of recording, an internal 16GB microSD card can hold roughly 4 hours of full HD (1080p, 30fps) video, and the weight is only 123g. The camera is equipped with two laser pointers mounted on the lens assembly, so the field of view can be checked and easily aligned, regardless of the camera’s mounted position, simply by rotating the lens assembly. The camera is mounted on the head with a band and is focused on the surgical field so that the camera will record whatever is in the surgeon’s view (Figure 1). The ContourHD system can be purchased online for less than 300 US dollars. The obtained digital data can be transferred to computer via USB cable and recorded videos can be easily edited using popular software (e.g. Adobe Premiere Elements 7.0, Adobe Systems, California, USA).

### Patients and recorded video

We recorded surgeries at 1080p, 30 fps, as MOV files. At conferences, where HD video projection equipment and large screens may be available, the captured videos can provide an audience with a nicely detailed and high-quality experience. Unfortunately, the size of high-resolution video files makes uploading them to limited data capacity websites problematic. Therefore, we re-sized and re-encoded our surgery videos as 360p MPEG-2 files for this paper. We have provided videos of surgeries recorded using this method after obtaining informed consent from the patients, including an emergency thoracotomy in the emergency room for chest stab wounds and a crush laparotomy for a severe liver injury, procedures that typically are difficult to record.

### Case 1: Emergency thoracotomy in the emergency room with pericardium incision

**Figure 2:**
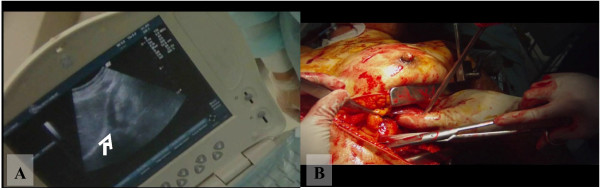
**Emergency thoracotomy in the emergency room with pericardium incision. A:** Subxiphoid view of bedside echocardiography performed by surgeon equipped with a head-cam. The presence of pericardial fluid (white arrow) enabled the diagnosis of cardiac tamponade to be made (video image capture). **B:** Left anterior thoracotomy and longitudinal incision over the pericardium made in the emergency department. The image shows significant hemorrhaging from the pericardium (video image capture).

A 46-year-old woman sustained multiple stab wounds in the bilateral chest. On admission, she was agitated, with a systolic pressure of 78 mmHg, pulse of 121/min, and oxygen saturation of 90% on face mask. She was intubated and initial resuscitation was started immediately. The focused assessment with sonography for trauma was positive for pericardial fluid (see Figure 2A, Additional file 1). Despite fluid resuscitation with 1500 ml of lactated Ringer’s solution, her condition deteriorated rapidly and she became pulseless, with a diagnosis of possible cardiac tamponade. A left anterior thoracotomy and a longitudinal incision over the pericardium were made, with care taken to avoid the phrenic nerve (see Figure 2B, Additional file 1). The findings were that of a massive hemopericardium, likely from a distal coronary artery, causing the tamponade. Coronary bleeding could be controlled with manual astriction and the incision was extended as a clamshell incision for detecting bilateral hemorrhagic spots and cardiac injury. She became hemodynamically stable and was then transferred to the operation room for definitive hemostasis. The patient subsequently improved and was discharged on the ninth day after surgery.

### Case 2: Crush laparotomy and hemostasis for a severe liver injury in multiple trauma

**Figure 3:**
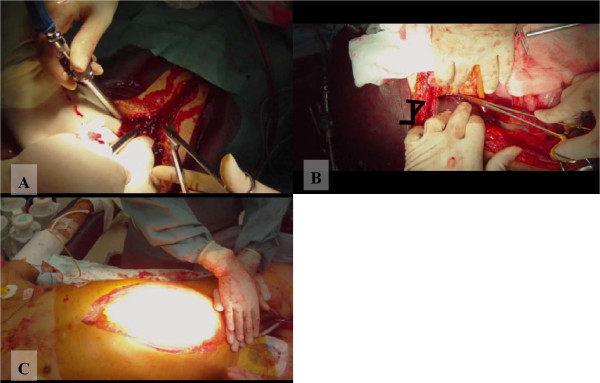
**Crush laparotomy and hemostasis for a severe liver injury in multiple trauma. A:** Crush laparotomy, showing significant hemorrhaging from the abdominal cavity (video image capture). **B:** Felt pledgeted sutures placed across the liver laceration site (black arrow) (video image capture). **C:** Temporary abdominal closure using the vacuum pack technique (video image capture).

A 26-year-old man was hit by a train and arrived at the emergency room with a systolic pressure of 72 mmHg and pulse 142/min. He had a grossly distended abdomen, and a bilateral lower thigh fracture. He was intubated and initial resuscitation was started immediately. After administration of 2000 ml of lactated Ringer’s solution, his systolic blood pressure was 98 mmHg. A computed tomography (CT) scan, taken after hemodynamic stabilization, showed an extravasation of contrast material from the right lobe of the liver, a facial fracture, and a pelvic fracture. Soon after the CT scan, his hemodynamic status deteriorated again, with drop in systolic blood pressure to 68 mmHg. At crush laparotomy, 3 L of blood was found, with lacerations of the right lobe and left lateral segment of the liver (see Figure 3A, Additional file 2). We decided on damage control surgery for the strategy because intraoperative findings showed massive hemoperitoneum with coagulopathy and physiologic abnormality, and the patient had sustained multiple trauma injuries. Felt pledgeted sutures, which help preserve the liver parenchyma, were placed across the site of the liver laceration (see Figure 3B, Additional file 2) using Pringle’s maneuver, clamping the hepatoduodenal ligament to interrupt the flow of blood through the hepatic artery and the portal vein and thus helping to control bleeding from the liver (see Additional file 3). Vacuum assisted closure was done for temporary abdominal closure after perihepatic packing (see Figure 3C, Additional file 3). Post operative angioembolization was performed to stop the bleeding of the pelvic and liver injuries and the facial fracture. Thereafter, the patient was given multiple surgeries and returned home on postoperative day 113.

## Discussion

The details of surgical techniques and a patient’s clinical status are hard to convey in writing, even when figures and photographs are included, and this is especially pertinent concerning intricate surgical handwork and hemorrhagic status. Current advances in video technology have enabled surgeons to make recordings of their operations and document procedures; these recordings can later be used for teaching, research, auditing, and patient education [4-6]. High-quality surgical videos have the following merits as educational tools: 1) images are clear and sharp, 2) videos are taken from the point of view of the operator, 3) clinically necessary intraoperative findings can be pointed out and explained, and 4) the zoom function allows for up-close and detailed recording of surgical procedures and anatomical features. Surgical videos thus offer numerous benefits, including not only in simulation training for surgical techniques, but also by enabling role-playing methods, to elucidate decision making concerning treatment strategies. Several authors have reported that the use of video can be an effective method for acquiring skill and for team debriefing in trauma resuscitation [8-10]. However, education using video may be more effective when combined with simulated trauma education, such as in ATLS (advanced trauma life support) scenarios. In addition, these videos can be used as medical time records to aid the development of new and efficient procedures. In recent years, a number of conferences have included sessions where videos of surgical procedures are shown, and many journals offer videos on their websites. Major organizations such as the American College of Surgeons and the Society of American Gastrointestinal and Endoscopic Surgeons already have video libraries that cover a multitude of surgical subjects and procedures. Moreover, the use of surgical videos as an educational tool is likely to become more widespread in the future.

Laparoscopic video is easy to record but capturing video during open surgeries is challenging. Although there are currently many options for recording surgery fields [4-7,11], none has previously been suitable for trauma surgery or emergency procedures in extremely busy scenarios. Operating light mounted cameras, which many hospitals use, or commercially available video cameras mounted on a tripod, is easy, but the view obtained by such equipment is apt to be blocked by the surgeon’s head, and capturing footage of deep body cavities is problematic. Although open surgery recording using laparoscopic cameras is superior to that when fixed cameras are used, in terms of acquiring a technical view of the surgical field, the successful use of such equipment requires bothersome preparation of a table-mounted holder or a dedicated camera operator [4].

Several medical head-cams are currently on the market and can accurately record surgeons’ views, but because these are connected to a video monitor, surgeon mobility is restricted and equipment preparation is usually required. As a result, this type of equipment is unsuitable for recording emergency procedures such as an emergency thoracotomy in the emergency room. In addition, medical equipment built for hospital-use tends to be very costly, but the rapid pace of technological development means that such equipment may soon become outdated, reducing the value of significant capital expenditures. Therefore, the simplest and most cost-effective method is to employ a commercially available item [12].

Commercially available head-cams without a video monitor are inexpensive and allow quick and easy recording of trauma surgeries. Also, in addition to the advantages offered by head-cams during emergency in-hospital procedures, they can be useful during the provision of pre-hospital care. However, for recording video during surgical procedures, commercially available head-cams have two major problems.

The first problem is that the view of the operator and that of the camera may be different and since no monitor or display is used, there is no way to verify the congruence of these views. The ContourHD model described in this paper has a built-in pair of laser pointers that indicate the plane of the camera’s view, but this feature is only used temporarily, to orient the camera lens appropriately prior to the start of recording. The second problem is that these head-cams operate in a fixed mode, with no zoom function. Therefore, although a general view is effectively captured, providing closer views of detailed procedures requires the use of video editing software that has a zoom function. However, new devices are being developed to mitigate these problems.

Decision making is very difficult when attending to major trauma [13]. The attending surgeon has a split second in which strategy must be decided, to undertake damage control surgery or early total care, by referring to physiological abnormalities and organ injuries. In conferences, written content concerning physiological abnormalities can be examined at leisure, but evaluation of detailed organ injuries requires images. Damage control surgery should have limited aims, namely, control of hemorrhaging and contamination, so simple techniques (suturing, resection, or packing) suffice, and intricate procedures are not required [14]. Therefore, commercially available head-cams can be extremely useful for recording general intraoperative findings and the simple procedures used in trauma surgery. If intricate procedures beyond damage control surgery are needed in trauma surgery, the situation is likely amenable to the use of other specialized recording devices.

## Conclusion

We described a simple and cost-effective method of recording trauma surgery. Although many options for recording surgical fields are available, each has its distinctive characteristics, advantages, and disadvantages. Recording devices that best meet the needs of the surgical situation should be chosen.

### Consent

Written informed consent for publication of the case report details and accompanying audiovisual material was obtained from all patients, copies of which are available for review by the Editor-in-Chief of this journal.

This report was presented at World Trauma Congress, August 2012 in Rio de Janeiro. This report has neither been published nor is it being considered for publication elsewhere.

This report has neither been presented nor is it being considered for publication elsewhere.

## Competing interests

The authors declare that they have no competing interests.

## Authors’ contribution

S.M conducted literature searches and conceived of this technique. S.M wrote the manuscript, which was critically revised by K.S and M.K. All authors read and approved the final manuscript.

## Supplementary Material

Additional file 1 Emergency thoracotomy in the emergency room with pericardium incision. Click here for file

Additional file 2 Crush laparotomy, showing significant hemorrhaging from the abdominal cavity. Click here for file

Additional file 3 Pringle’s maneuver and vacuum assisted closure after perihepatic packing. Click here for file
